# Diagnosis of atypical carcinoid can be made on biopsies > 4 mm^2^ and is accurate

**DOI:** 10.1007/s00428-022-03279-7

**Published:** 2022-01-28

**Authors:** Ellen M. B. P. Reuling, Dwayne D. Naves, Johannes M. A. Daniels, Chris Dickhoff, Pim C. Kortman, Mark A. M. B. Broeckaert, Peter W. Plaisier, Erik Thunnissen, Teodora Radonic

**Affiliations:** 1 Department of Surgery, Amsterdam University Medical Center, VU University Amsterdam, Amsterdam, the Netherlands; 2 Department of Surgery, Albert Schweitzer Hospital, Albert Schweitzerplaats 25, 3318 AT Dordrecht, the Netherlands; 3 Department of Pathology, Amsterdam University Medical Center, VU University Amsterdam, De Boelelaan 1117, 1081 HV Amsterdam, the Netherlands; 4 Department of Pulmonary Diseases, Amsterdam University Medical Center, VU University Amsterdam, Amsterdam, the Netherlands; 5 Department of Cardiothoracic Surgery, Amsterdam University Medical Center, VU University Amsterdam, Amsterdam, the Netherlands; 6 Cancer Centre Amsterdam, De Boelelaan 1117, 1081 HV Amsterdam, the Netherlands

**Keywords:** Typical carcinoid, Atypical carcinoid, Biopsy size, Endobronchial therapy Ki-67

## Abstract

In the 2021 WHO thoracic tumors, gradation of lung carcinoids in biopsies is discouraged. We hypothesized that atypical carcinoid (AC) could be reliably diagnosed in larger preoperative biopsies. Biopsy-resection paired specimens of carcinoid patients were included, and definitive diagnosis was based on the resection specimen according to the WHO 2021 classification. A total of 64 biopsy-resection pairs (26 typical carcinoid (TC) (41%) and 38 AC (59%)) were analyzed. In 35 patients (55%), tumor classification between the biopsy and resection specimen was concordant (26 TC, 9 AC). The discordance in the remaining 29 biopsies (45%, 29 TC, 0 AC) was caused by misclassification of AC as TC. In biopsies measuring < 4 mm^2^, 15/15 AC (100%) were misclassified compared to 14/23 AC (61%) of biopsies ≥ 4 mm^2^. Categorical concordance of Ki-67 in biopsy-resection pairs at threshold of 5% was 68%. Ki-67 in the biopsy was not of additional value to discriminate between TC and AC, irrespective of the biopsy size. Atypical carcinoid is frequently missed in small bronchial biopsies (< 4 mm^2^). If the carcinoid classification is clinically relevant, a cumulative biopsy size of at least 4 mm^2^ should be considered. Our study provides strong arguments to make the diagnosis of AC in case of sufficient mitosis for AC on a biopsy and keep the diagnosis “carcinoid NOS” for carcinoids with ≤ 1 mitosis per 2 mm^2^. Ki-67 has a good concordance but was not discriminative for definitive diagnosis.

## Introduction

Pulmonary carcinoids comprise a subgroup of neuroendocrine tumors and are categorized into low-grade typical carcinoid (TC) and intermediate-grade atypical carcinoid (AC) according to the current WHO classification [1]. Morphologically, TC is defined as a neuroendocrine tumor with 0 or 1 mitoses per 2 mm^2^and absence of necrosis, while AC has 2–10 mitoses per 2 mm^2^and/or dot-like necrosis [2, 3]. Ki-67 is a widely accepted marker in the diagnostic pathology of gastrointestinal neuroendocrine tumors [4] and it showed a lower interobserver variability than the mitotic count [5]. However, Ki-67 is currently not used for distinction between TC and AC, but some literature and expert opinion in the current 2021 WHO classification suggest that a Ki-67 ≥ 5% might be suggestive of AC [1, 6]. Accurate identification of AC at time of diagnosis can be clinically relevant as it directs treatment selection. For example, endobronchial treatment is a promising parenchyma sparing procedure for selected patients with centrally growing intraluminal bronchial TC. During this parenchyma-sparing procedure, a rigid bronchoscope is used which allows for larger biopsies and in selected cases even complete resection [7]. Furthermore, diagnostic accuracy might implicate a more aggressive search for potential dissemination as AC tend to metastasize more often than TC [3]. However, in the latest WHO classification of thoracic tumors 2021, classification of carcinoids in the biopsy is discouraged, suggesting a diagnostic term “carcinoid NOS” in the biopsy [1]. We hypothesized that AC could be reliably diagnosed in larger preoperative biopsies. To test this hypothesis, we investigated the relation of biopsy surface, Ki-67, and accuracy of diagnosing of AC correctly.

## Methods

Approval of the institutional review board (Medical Ethics Review Committee of VU University Medical Center, IRB00002991) was retrieved. Patients who underwent surgical resection for centrally located pulmonary carcinoid (stages I–III) between June 1991 and December 2019 at the Amsterdam University Medical Center were screened for eligibility. Central tumors were defined as tumors situated proximal to the segmental bronchi. Patients who had paired diagnostic biopsies obtained with either flexible (FLB) or rigid (RIB) biopsy were selected. Samples of central carcinoid tumors were independently evaluated by two pathologists (TR and ET) and scored for mitotic count, presence of necrosis, and diagnosis. The Ki-67 scoring was based on an estimated percentage of positive cells in a hotspot region after scanning the whole slide. Ki-67 index was calculated in surgical specimens by counting at least 2000 consecutive tumors cells in hot spot fields at × 40 magnification or 2 mm^2 ^for consistency with the histological classification [6]. The highest recorded value was taken into account, as described before [8]. Two Ki-67 thresholds of 3 and 5% described in the literature were used [6, 9, 10]. Tumor classification on the resection specimen was considered as the gold standard, and thus marked as definitive diagnosis. Mitotic figures on biopsies and resection specimen were counted as described previously [11]. In short, the whole slide was first explored for mitotic hotspots and the mitotic count was subsequently performed in the hotspot area. HE-stained slides were scanned using Phillips UFS scanner and analyzed with the Philips pathology viewer version 3.2. Histological tumor sample size was digitally measured and defined as tumor surface (mm^2^) in the whole histological sample. Areas with cauterization or mechanical artifacts were discarded. The statistical analyses and calculations were performed with IBM SPSS Statistics for Windows, version 26 (IBM Corp., Armonk, N.Y., USA) (ER, DN).

### Results

Paired biopsy and resection specimens of central pulmonary carcinoids from 64 patients were available. The diagnosis was based on mitotic count, as (dot-like) necrosis was absent. No significant differences were observed between patients with TC (*n* = 26) and AC (*n* = 38) regarding clinic-pathological characteristics, except for a trend of a larger tumor diameter in patients with AC (*p* = 0.05) (Table 1).

**Table 1:** Clinicopathological characteristics of central carcinoid cohort

Patient characteristics	*N* = 64 (%)	TC (*n* = 26)	AC (*n* = 38)	*p*-value
Mean age in years (SD)	45 (16.2)	43 (15.8)	46 (16.7)	0.51
Female	40 (62.5)	14 (53.8)	25 (65.8)	0.33
Biopsy				
FLB	34 (53.1%)	12 (46.2%)	22 (57.9%)	0.36
RIB	30 (46.9%)	14 (53.8%)	16 (42.1%)	
Type of surgery	64	26	38	0.10
Pneumonectomy	3 (4.7)	2 (7.7)	1 (2.6)	
Bilobectomy	13 (20.3)	9 (34.6)	5 (13.2)	
Lobectomy	29 (45.2)	8 (30.7)	20 (52.7)	
Sleeve lobectomy	17 (26.6)	6 (23.1)	11 (28.9)	
Segmentectomy	1 (1.6)	1 (3.8)	0	
Bronchial sleeve resection	1 (1.6)	0	1 (2.6)	
Tumor diameter				0.05
T1a	38 (59.4)	21 (80.8)	17 (44.7)	
T1b	14 (22)	3 (11.5)	11 (28.9)	
T2a	8 (12.5)	2 (7.7)	6 (15.8)	
T2b	2 (3.1)	0	2 (5.3)	
T3	2 (3.1)	0	2 (5.3)	
T4	0	0	0	
Nodal stage				0.78
N0	59 (92.2)	25 (96.2)	34 (89.5)	
N1	4 (6.3)	1 (3.8)	3 (7.9)	
N2	1 (1.6)	0	1 (2.6)	
Radicality				0.68
R0	58 (90.6)	23 (88.5)	35 (92.1)	
R1	6 (9.4)	3 (11.5)	3 (7.9)	

The diagnosis was concordant with definitive pathology in 35 out of 64 patients (55%, 26 TC, 9 AC). In the remaining 29 (45%) patients, the biopsy-based diagnosis was TC (*n* = 29), while the diagnosis in the pulmonary resection specimen was AC. If AC was diagnosed in the biopsy, the diagnosis was consistently accurate (9/9, 100%; Table 2). In biopsies measuring < 4 mm^2^, 15/15 AC (100%) were misclassified as TC and in biopsies ≥ 4 mm^2^, 14/23 (61%) AC were misclassified as TC (Table 2). Accuracy of correctly identifying AC in the biopsy did not further increase with the biopsy diameter (data not shown).

**Table 2:** Diagnosis in the biopsy versus resection specimen, stratified for the biopsy size < and ≥ 4 mm^2^ and stratified for Ki-67 < and ≥ 3 (B) and 5% (C). Note that none of the AC could be diagnosed in biopsies < 4 mm^2^ and when AC was diagnosed on the biopsy, it was always concordant with the resection specimen (underscored A). Ki-67 was not of additional value for discrimination between TC and AC in the biopsy (underscored B and C). Biopsy size combined with a Ki-67 < and ≥ 5% did not increase diagnostic accuracy (D). *Ki-67 missing (*n* = 1)

	Diagnosis resection		
	TC	AC	Total
A			
Diagnosis TC biopsy < 4mm^2^	6	15	21
Diagnosis TC biopsy ≥ 4mm^2^	20	14	34
Diagnosis AC biopsy < 4mm^2^	0	0	0
Diagnosis AC biopsy ≥ 4mm^2^	0	9	9
	26	38	64
B			
Diagnosis TC biopsy Ki-67 < 3%	17	20	37
Diagnosis TC biopsy Ki-67 ≥ 3%	9	8	17
Diagnosis AC biopsy Ki-67 < 3%	0	4	5
Diagnosis AC biopsy Ki-67 ≥ 3%	0	5	5
	26	37	63*
C			
Diagnosis TC biopsy Ki-67 < 5%	18	23	42
Diagnosis TC biopsy Ki-67 ≥ 5%	8	5	12
Diagnosis AC biopsy Ki-67 < 5%	0	5	5
Diagnosis AC biopsy Ki-67 ≥ 5%	0	4	4
	26	37	63*
D			
Biopsy < 4mm^2^ and Ki-67 < 5%	5	13	19
Biopsy < 4mm^2^ and Ki-67 ≥ 5%	1	1	2
Biopsy ≥ 4mm^2^ and Ki-67 < 5%	13	15	28
Biopsy ≥ 4mm^2^ and Ki-67 ≥ 5%	7	8	15
	26	37	63*

The Ki-67 proliferation index was assessed with cut-off values of 3 and 5% in the biopsy. At a cut off of < 3% in the biopsy, 20/37 (54%) AC’s where misclassified as TC, compared to 8/17 (47%) ≥ 3%.In biopsies with a cut-off value of < 5%, 23/42 (55%) of the AC’s where misclassified as AC compared to 5/12 (42%) in biopsies of Ki-67 ≥ 5% (Table 2B and C). In biopsies > 4 mm^2^, Ki-67 did not increase the diagnostic accuracy for TC or AC (Table 2D).

**Fig. 1:**
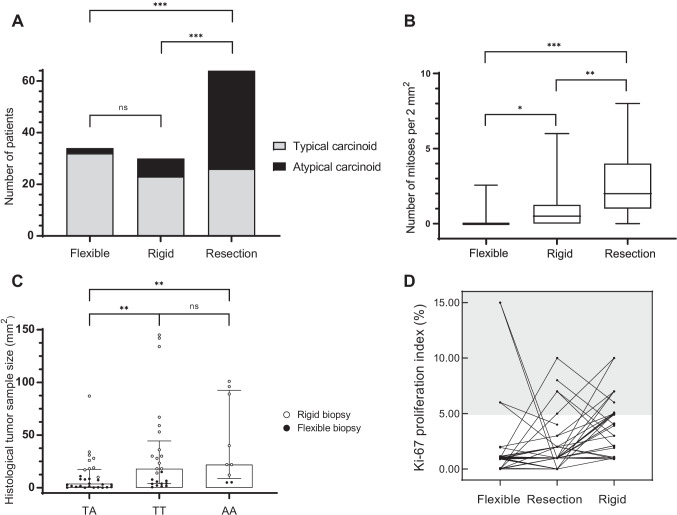
Outcomes in flexible biopsy, rigid biopsy, and resection in relation to typical and atypical carcinoid (**A**) and mitotic count (**B**). Discordancy and concordancy between biopsy and resection in relation to biopsy sample size; discordant TA: diagnosis in biopsy TC and in resection AC; concordant TT: biopsy and resection diagnosis TC; AA: biopsy and resection diagnosis AC (**C**). Concordancy between biopsy (flexible or rigid) and resection in relation to Ki-67 (**D**). *p ≤ 0.05; **p ≤ 0.01; ***p ≤ 0.001

Figure 1 presents the distribution of diagnoses (TC vs AC), mitotic count and Ki-67 index for flexible biopsy (FLB), rigid biopsy (RIB), and surgical resection specimen respectively. When considering biopsies obtained with FLB and RIB separately, discordance was 59% and 30%, respectively (*p* = 0.021). In total, 38 (59%) cases were identified with definitive AC diagnosis. Nine histological AC diagnoses were made in the biopsies, more often in RIB (7/30, 23%) than in FLB (2/34, 6%) (*p* = 0.07, Fig. 1A). In RIB, a higher number of mitotic figures were demonstrated when compared with FLB (*p* = 0.012). In addition, RIB were significantly larger than FLB (median histological tumor sample size FLB; 3.1 mm^2^(range 0.1–15 mm^2^) vs RIB; 29 mm^2^, (range 10–145 mm^2^), *p* < 0.001).

Concordance in diagnosis between biopsy and resection was associated with increasing biopsy surface area (median sample size biopsy TA (diagnosis in biopsy TC and in resection AC) 3.9 mm^2^(range 0.10–87 mm^2^), TT (biopsy and resection diagnosis TC) 18 mm^2^(range 0.6–145 mm^2^), AA (biopsy and resection diagnosis AC) 22 mm^2^(range 5–101 mm^2^)). Discordant biopsies versus concordant biopsies for typical carcinoid (*p* = 0.009) and atypical carcinoid (*p* = 0.004) where significantly smaller (Fig.1C). In 68% (43/63) of the cases, the Ki-67 in the biopsy and resection were concordant (concordance category 1: Ki-67 0–5%, concordance category 2: Ki-67 ≥ 5%), more often in FLB (26/33, 79%) than in RIB (17/30, 57%) (*p* = 0.05, Fig.1D).

## Discussion

In the current study, we showed that, the diagnosis of AC could be made on the biopsy and if so, the diagnosis was always accurate. Moreover, AC was consistently missed in biopsies < 4 mm^2^. Biopsies < 4mm^2^, all taken during flexible bronchoscopy, resulted in 59% of the patients discordantly classified as TC, compared to 30% of biopsies obtained with rigid bronchoscopy. Ki-67 in the biopsy did not show additional value in the discrimination between TC and AC, irrespective of the biopsy size.

Even though the classification of carcinoids in the biopsies is discouraged in the recent WHO 2021 [1], there are clinical situations where identification of AC in the biopsy might be relevant for the treatment choice [12–17]. For example, EBT and parenchyma saving procedures are not preferred in patient with AC. Our data showed that if AC could be diagnosed on a biopsy, the diagnosis was consistently accurate. However, out study also shows AC may be missed, even in larger biopsies. A classification as TC on a biopsy should be interpreted with caution and a diagnosis of “carcinoid NOS” is more appropriate. The current WHO classification suggests that if the diagnosis AC can be made, this “may be suggested in a comment.” Our study provides strong arguments to make the diagnosis of AC in case of sufficient mitosis for AC and keep the diagnosis “carcinoid NOS” for carcinoids with ≤ 1 mitosis per 2 mm^2^.

**Fig. 2:**
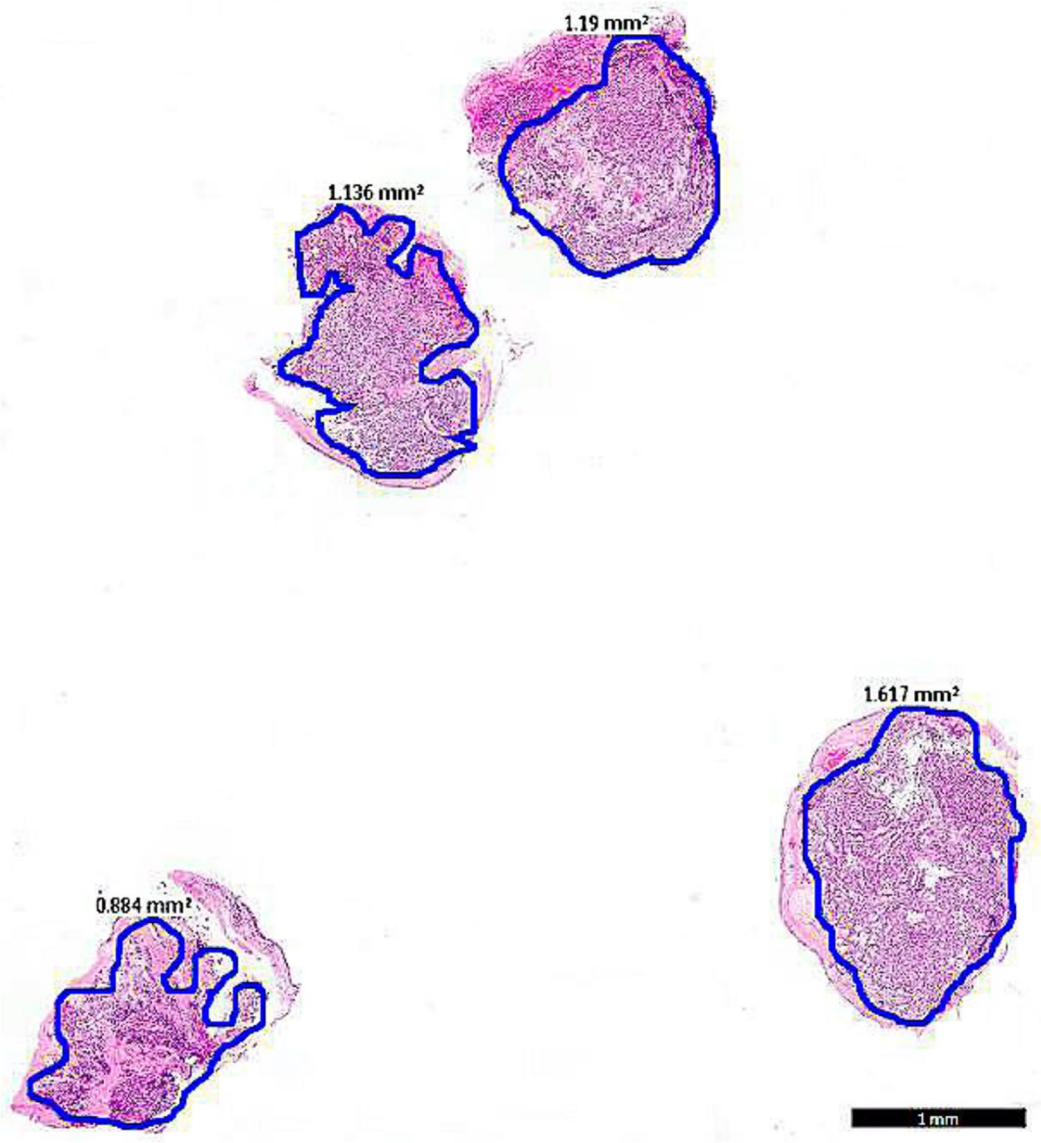
Example of 4 flexible biopsies of 1 mm^2^ with bronchial carcinoid

Our data are largely in line with a recent retrospective study analyzing the accuracy of pre-operative biopsies for bronchial carcinoid tumors. The authors reported a 57% discrepancy when diagnosis in the biopsy was compared to postoperative diagnosis with a wider variety of discrepancies [18]. In contrast to our data, only 15/330 (4.5%) AC’s were diagnosed in the biopsy of which 6 were reclassified as TC in the resection specimen. However, this real-life study was based on the pathology reports of the national database. In our in depth study of biopsy type and diameter, we provide at least the partial explanation of the discordances in biopsy-resection pairs. Although underdiagnoses of AC were not excluded in larger biopsies, AC was always missed in biopsies < 4 mm^2^. Conceptually, a preferred cumulative biopsy surface may be estimated that is associated with a higher diagnostic accuracy. We assume that a cumulative surface of 4 mm^2^ is equivalent to ± 4 bronchial biopsies of 1 mm^2^ tumor (Fig. 2) or 2 biopsies of 2 mm^2^ tumor. Therefore, if a preoperative identification of AC patients is of clinical importance, biopsy of ≥ 4 mm^2^ should be considered.

This study could not show any additional value of Ki-67 proliferation index in the biopsy for discrimination of TC and AC in the resection, comparable to some previously published studies [10, 19]. Published evidence so far has not allowed for definitive cut-off points to be determined for Ki-67 [6, 10], although the 5^th^ edition WHO guideline for thoracic tumors currently suggest that a Ki-67 ≥ 5% is most probably an AC [1]. Therefore, usage of Ki-67 as a diagnostic tool in lung carcinoid might be still debatable. However, Ki-67 might be useful as an independent prognostic marker of dissemination, additional to the mitotic count [6]. Current study did not investigate this aspect of Ki-67. Interestingly, concordance in Ki-67 between the biopsy and resection was higher than for the diagnosis TC or AC (based on the mitotic count), similar as described before [6]. Remarkably, flexible biopsies showed a higher Ki-67 concordance with the resection than rigid biopsies (79% vs 59% respectively). This might be explained by the high rate of tumor debulking during the rigid bronchoscopy (endobronchial treatment) and only a small tumor rest in the resection. Concordance of 79% is therefore probably more reflective of the daily clinical practice worldwide.

Flexible biopsies are easier to obtain and require less sedation compared to rigid biopsies. However, performing 4 biopsies in a relatively high vascularized tumor in non-anesthetized patients is challenging due to a risk of difficult bleeding control. Preferably these biopsies should be performed in a controlled setting under general anesthesia via a rigid bronchoscopy in specialized centers and should be considered whenever clinically relevant, e.g., in patients with central bronchial carcinoid tumors suitable for curative endobronchial therapy, or patients unfit for surgery in whom bronchoscopic debulking could relieve symptoms of dyspnea or post-obstructive pneumonia.

Thus, biopsy size does matter, which was previously shown in large cell neuroendocrine carcinoma, where neuroendocrine morphology was more frequently lacking in smaller biopsies (< 5 mm) when compared to larger biopsies [20]. In addition, for determination of PD-L1 in lung cancer, a biopsy size of < 2 mm is associated with a 14% chance of false negative score [21, 22]. These examples, and findings from the current study, underscore the fact that small biopsy samples are associated with “false negatives”/underdiagnoses.

In our cohort, a larger proportion of AC was observed than in the literature [3, 15]. A possible explanation may be a selection bias, as our center is a tertiary referral center for endobronchial treatment and more complex surgery. The higher proportion of AC allowed a more precise investigation of diagnostic accuracy for AC in a relatively small patient population, the latter being a potential limitation of this study.

## Conclusion

The diagnosis AC is frequently missed in small bronchial biopsies (< 4 mm^2^). If the carcinoid classification is clinically relevant, a cumulative biopsy surface of at least 4 mm^2^ should be considered. Our study provides strong arguments to make the diagnosis of AC in case of sufficient mitosis for AC on a biopsy and keep the diagnosis “carcinoid NOS” for carcinoids with ≤ 1 mitosis per 2 mm^2^. Ki-67 in biopsy-resection pairs had a higher concordance but was of no additional discriminative value for the definitive diagnosis, irrespective of the biopsy size.

## Data Availability

The data that support the findings of this study are available from the corresponding author EMBP Reuling. Restrictions apply to the availability of these data, which were used under license for this study.
