# Correction to: Diagnosis of atypical carcinoid can be made on biopsies > 4 mm^2^ and is accurate

**DOI:** 10.1007/s00428-022-03294-8

**Published:** 2022-02-07

**Authors:** Ellen M. B. P. Reuling, Dwayne D. Naves, Johannes M. A. Daniels, Chris Dickhoff, Pim C. Kortman, Mark A. M. B. Broeckaert, Peter W. Plaisier, Erik Thunnissen, Teodora Radonic

**Affiliations:** 1 Department of Surgery, Amsterdam University Medical Center, VU University Amsterdam, Amsterdam, the Netherlands; 2 Department of Surgery, Albert Schweitzer Hospital, Albert Schweitzerplaats 25, 3318 AT Dordrecht, the Netherlands; 3 Department of Pathology, Amsterdam University Medical Center, VU University Amsterdam, De Boelelaan 1117, 1081 HV Amsterdam, the Netherlands; 4 Department of Pulmonary Diseases, Amsterdam University Medical Center, VU University Amsterdam, Amsterdam, the Netherlands; 5 Department of Cardiothoracic Surgery, Amsterdam University Medical Center, VU University Amsterdam, Amsterdam, the Netherlands; 6 Cancer Centre Amsterdam, De Boelelaan 1117, 1081 HV Amsterdam, the Netherlands

**Correction to: Virchows Archiv**

10.1007/s00428-022-03279-7

In the original version of this article, the author name **E Thunnissen** should have been written as **Erik Thunnissen.**

The original article has been corrected.

